# Domestic

**Published:** 1841-11-20

**Authors:** 


### DOMESTIC.

### HEALTH OF THE CITY.

INTERMENTS *in the City and Liberties of Philadelphia, from the 6th to the 13th of November.*

| Diseases. | Adults. | Children. |
|---|---|---|
| Abscess Lumbar, | 1 | 0 |
| Apoplexy | 1 | 0 |
| Croup, | 0 | 4 |
| Congestion of brain, | 0 | 2 |
| Consumption of the lungs, | 13 | 3 |
| Convulsions, | 2 | 7 |
| Diarrhœa, | 0 | 1 |
| Dropsy, | 4 | 0 |
| —— Abdominal, | 1 | 0 |
| —— Head, | 0 | 1 |
| —— Breast, | 1 | 1 |
| Disease of the heart, | 1 | 0 |
| Drowned, | 1 | 0 |
| Dysentery, | 2 | 0 |
| Debility | 0 | 1 |
| Fever, congestive, | 1 | 0 |
| —— Bilious, | 0 | 1 |
| —— Nervous, | 1 | 0 |
| —— Typhoid, | 1 | 0 |
| —— Scarlet, | 0 | 1 |
| Inflammation of Bronchi, | 0 | 2 |
| —— Lungs, | 1 | 1 |
| —— Heart, | 1 | 0 |
| Ileus, | 1 | 0 |
| Intemperance, | 2 | 0 |
| Inanition, | 0 | 1 |
| Marasmus, | 0 | 3 |
| | — | — |
| *Carried forward,* | 35 | 29 |

| Diseases. | Adults. | Children. |
|---|---|---|
| *Brought forward,* | 35 | 29 |
| Menorrhagia, | 1 | 0 |
| Old age, | 6 | 0 |
| Palsy, | 1 | 0 |
| Scrofula, | 0 | 2 |
| Small pox, | 6 | 6 |
| Still-born, | 0 | 6 |
| Spitting of blood, | 1 | 0 |
| Ulceration of throat, | 0 | 1 |
| Varioloid, | 0 | 1 |
| Violence, | 0 | 2 |
| | — | — |
| Total, 97— | 50 | 47 |

Of the above, there were under 1 year, 28

| | |
|---|---|
| From 1 to 2 | 3 |
| 2 to 5 | 9 |
| 5 to 10 | 4 |
| 10 to 15 | 2 |
| 15 to 20 | 1 |
| 20 to 30 | 14 |
| 30 to 40 | 9 |
| 40 to 50 | 9 |
| 50 to 60 | 5 |
| 60 to 70 | 6 |
| 70 to 80 | 5 |
| 80 to 90 | 1 |
| 90 to 100 | 1 |
| Total, | 97 |

Of the above there were 10 from the almshouse, and 9 people of colour, which are included in the total amount.

*Zabriskie on Paralysis from Visceral Disorders.*

From this paper, we extract the following conclusions:

The pathology of paraplegia has been the subject of much argumentation. The paralysis of the lower extremities was formerly considered as depending upon a diseased state of the spinal cord or nerves; but, of late, many distinguished physiologists have contended that it originated in some part of the brain. Baillie, Abercrombie, Cooke and others, contend for this position. There can be no doubt, however, at present, that paraplegia often arises from both causes—as dissection has discovered disease in some cases in the brain, in others in the spine. But as there are cases in which no morbid appearances have been discovered in the brain, spine, or in the nerves, we are led to look for the seat of the disease in some other part. There is a very marked distinction between the symptoms of paralysis as proceeding from a morbid state of the brain and from other causes. Dr. Baillie observes, that there is always more or less disturbance of the intellectual faculties; the memory is impaired, and the organs of the senses are deficient in power. Frequently the sight of one eye is lost; the tongue becomes feeble in its motion. Dr. Calmeil, who examined a great many cases of this kind, considers it a thing impossible that the reason should long remain untouched in general paralysis where the brain is profoundly affected.

An extremely interesting memoir upon this subject will be found in the American Journal of Medical Sciences, (Feb. 1835, p. 299,) by Dr. Waddel, of Tallahassee, who relates a case of paraplegia in which there was a complete paralysis of the lower half of the body, attended with symptoms of diseased bowels. Dissection showed the spine and its contents sound. The mucous membrane of the stomach and intestines was highly inflamed, and the intestines contained a yellow pus. The duodenum was perforated by an ulceration half an inch in diameter.

Dr. Waddel considers this disease as residing in the sympathetic nerve, its branches and ganglia. This memoir shows that he has carefully examined the subject, and his arguments are very ingenious. He supposes the nervous system of the intestines highly inflamed, and in consequence of its exalted state, the conducting capacity of the sympathetic nerve was so elevated by disease as to divert the nervous principle from its proper destination, and spend it upon the intestines. This argument, though ingenious, is unfortunately not based upon facts and post mortem examinations.

In the very case which acts as the foundation of Dr. Waddel's argument, he could find no trace of inflammation of the sympathetic nerve; none was observed in the case of Ann Masters, mentioned above; and in all the cases recorded by Mr. Abernethy and others that I have been able to find, there was observed no morbid state of the apparatus of the sympathetic nerve, while in every case there was found an inflammation or ulceration of the intestines. The very argument used by Dr. Waddel may be used against himself. For if it is fair to infer from the absence of all morbid appearances upon dissection in the brain, spinal cord or nerves, of the absence of any disease in these organs, it is equally fair to infer from the absence of all morbid appearances in the apparatus of the sympathetic nerve, of the absence of all disease in these organs.

Lobstein, in his interesting and learned work upon the sympathetic nerve, has described many cases of inflammation of the ganglia and of organic diseases of this nervous system, but there is only one case in which these morbid changes were attended with paralysis, and in that case there was caries of the vertebræ.

It is therefore much more rational to consider the disease as enteritis, the paraplegia as symptomatic of the visceral inflammation, and the sympathetic nerve through its spinal branches the means of communication of this morbid action to the spinal apparatus. As the cerebral and spinal nerves are the nerves of voluntary motion, and the office of the sympathetic is visceral, being chiefly concerned in producing involuntary or organic motion, it is not rational to suppose that a morbid state of the sympathetic nerve would cause an entire suspension of all the functions of the spinal nerves.

Besides, in all these cases, the hollow muscles supplied by the branches of the intercostal nerve were not paralyzed, but only the muscles of voluntary motion which are supplied by the spinal nerves.

The reasons for believing this species of paralysis as sympathetic of enteritis, may be briefly summed up as follows :—

1. The inflammation always precedes the paralysis, and often for some time. This took place in Dr. Waddel's case, in all the cases of Mr. Abernethy, and in all the cases observed by myself, the diarrhœa preceded the paraplegia.

2. From the absence of all morbid appearances upon dissection, the nervous apparatus appearing sound.

3. From the inutility of all remedies applied to the spine, to the brain, or to the general nervous system.

4. The remedies which gave most relief were those which relieved the inflammatory symptoms.

The diagnosis of this form of paraplegia may be attended with difficulty. But it may be distinguished from cerebral paralysis by the absence of all symptoms of diseased brain. From the paralysis arising from a disease of the spine, by the absence of the severe pain, and tenderness upon pressure in a diseased state of the vertebræ, by the existence of visceral inflammation, by the effect of remedies used for enteritis, and by the gradual manner of its approach.

The practice which I have found most successful, is that calculated to subdue the inflammatory symptoms.

Bleeding general and local to subdue the phlogosis, accompanied by evacuants, followed by mercurials when these symptoms abate, to change the morbid secretions, and, by counter-irritation, to divert the inflammation. When the inflammatory symptoms have somewhat subsided, strychnine forms an excellent and valuable remedy in paraplegia. Having used this substance very extensively, I would observe that it is of most use in paralysis which is symptomatic of visceral irritation. The nux vomica has been often used and highly recommended by European physicians in chronic dysentery and other diseases of the bowels, and therefore a part of the modus operandi of strychnine may be its effect upon the visceral disease; but it also certainly has an effect upon the nervous system independently of any remedial effect in enteritis. This is proved by its effect in cerebral and spinal paralysis. Its beneficial effects here, though not as manifest as in some other varieties, are in some cases very well marked, while in others no benefit can be perceived. In paralysis arising from the deleterious effects of lead, it is often a powerful remedy. In every case it should be given in small doses until its peculiar effects are produced upon the brain, when it should be discontinued for a short time until these effects have passed away, when it may be again administered if necessary.

The cases alluded to by the author are several in number, and in general followed dysentery. During the past summer an unusual number of such cases occurred to us, but there was little evidence of active inflammation; the patients were feeble, and more or less exhausted by the previous attacks, the paralysis occurring either at the termination of the dysentery, or nearly so. Blistering to the spine, frictions to the lower extremities, and, in some cases, cupping, were sufficient for a speedy cure.

